# Negative Mood Induction Increases Choice of Heroin Versus Food Pictures in Opiate-Dependent Individuals: Correlation With Self-Medication Coping Motives and Subjective Reactivity

**DOI:** 10.3389/fpsyt.2019.00274

**Published:** 2019-05-15

**Authors:** Lee Hogarth, Lorna Hardy, Alexandra Bakou, Justin Mahlberg, Gabrielle Weidemann, Sharon Cashel, Ahmed A. Moustafa

**Affiliations:** ^1^School of Psychology, University of Exeter, Exeter, United Kingdom; ^2^School of Social Sciences and Psychology, Western Sydney University, Sydney, NSW, Australia; ^3^MARCS Institute for Brain, Behaviour and Development, Western Sydney University, Sydney, NSW, Australia

**Keywords:** negative mood induction, coping motives, heroin-seeking behavior, opiate dependence, vulnerability

## Abstract

Acute growth in negative affect is thought to play a major role in triggering relapse in opiate-dependent individuals. Consistent with this view, three lab studies have demonstrated that negative mood induction increases opiate craving in opiate-dependent individuals. The current study sought to confirm these effects with a behavioral measure of heroin seeking, and test whether the effect is associated with self-reported opiate use to cope with negative affect and subjective reactivity to mood induction. Participants were heroin-dependent individuals engaged with treatment services (n = 47) and control participants (n = 25). Heroin users completed a questionnaire assessing reasons for using heroin: negative affect, social pressure, and cued craving. Baseline heroin choice was measured by preference to enlarge heroin versus food thumbnail pictures in two-alternative forced-choice trials. Negative mood was then induced by depressive statements and music before heroin choice was tested again. Subjective reactivity was indexed by negative and positive mood reported at the pre-induction to post-test timepoints. Heroin users chose heroin images more frequently than controls overall ( *p* = .001) and showed a negative mood-induced increase in heroin choice compared to control participants (interaction *p* < .05). Mood-induced heroin choice was associated with self-reported heroin use to cope with negative affect ( *p* < .05), but not social pressure ( *p* = .39) or cued craving ( *p* = .52), and with subjective mood reactivity ( *p* = .007). These data suggest that acute negative mood is a trigger for heroin seeking in heroin-dependent individuals, and this effect is pronounced in those who report using heroin to cope with negative affect, and those who show greater subjective reactivity to negative triggers. Interventions should seek to target negative coping motives to build resilience to affective triggers for relapse.

## Introduction

According to negative reinforcement theory, negative affective states act as powerful triggers for drug use behavior, motivating drug use to cope with those states [e.g., Refs. (1–3)]. Evidence for this proposal comes from lab studies where negative mood induction (including stress) increased various metrics of drug motivated behavior, including craving, choice, demand, consumption, and cognitive bias. Such mood induction effects have been extensively demonstrated for alcohol (4–7), tobacco (8–10), and cocaine (11–14). Furthermore, individual sensitivity to negative mood-induced craving predicts relapse in alcohol- (15–18) and cocaine-dependent individuals (19, 20), suggesting this sensitivity is an important risk factor for relapse.

Three studies have tested whether negative mood induction motivates opiate craving in opiate-dependent individuals. The study by Childress and colleagues (21) recruited 10 opiate-dependent clients who had been abstinent for 30 days, exposed them to guided self-hypnosis of a depressive scene, and found that subjective opiate craving increased from pre- to post-induction. The second study by Hyman and colleagues (22) recruited 14 opiate-dependent individuals who had been detoxified and were undergoing naltrexone treatment. Exposure to guided imagery of a personalized stress situation increased subjective opiate craving from baseline, while exposure to neutral imagery had no effect. Positive correlations were found between stress effects on craving and subjective reactivity (anxiety, fear, and sadness). Finally, the third study by Stathopoulou and colleagues (23) recruited 76 opiate-dependent individuals who had been on methadone maintenance for 4 months and exposed them to short video clips to induce sadness. After excluding 10 participants who showed no increase in subjective negative mood, it was found that the increase in craving from pre to post was related to subjective negative mood, and was moderated by anxiety such that this relationship was only significant in those with high anxiety sensitivity. There was no relationship between mood-induced craving and self-reported opiate use to cope with negative affect. Overall, this work provides preliminary support for the notion that acute negative mood is an important trigger for opiate seeking.

One limitation of the existing literature is that there is no demonstration of negative mood induction increasing a behavioral measure of heroin-seeking behavior. The three prior studies all measured opiate craving which has an unknown relationship to behavior (24). To address this limitation, we employed a pictorial choice procedure in which opiate-dependent individuals had the choice to enlarge heroin versus food thumbnail pictures in a series of two-alternative forced choice trials. Prior studies have validated the pictorial drug choice task by demonstrating that percent drug choice was increased in drug users versus non-users, or as a function of dependence level in the drug user group, in cocaine (25, 26), alcohol (27, 28), and tobacco users (28, 29), and was sensitive to the motivating effects of negative mood induction (10, 27, 30). In the current study, opiate users and control participants completed a concurrent pictorial choice task for heroin versus food pictures before and after mood induction. The first prediction was that heroin users would choose heroin images more frequently than control participants, validating the pictorial choice measure as an index of heroin value. The second prediction was that heroin users would show a mood-induced increase in heroin choice whereas control participants would not, suggesting that acute negative affect is an important trigger for heroin-seeking behavior.

The second limitation of the existing literature is that individual sensitivity to mood-induced opiate craving remains obscure. The two studies by Hyman et al. and Stathopoulou et al. (22, 23) found that mood-induced opiate craving was associated with subjective mood reactivity, consistent with a range of other induction studies [e.g., Refs. (27, 31–34), but see Refs. (35, 36)]. Consequently, the third prediction of the study was that mood-induced heroin choice would be associated with subjective mood reactivity. More interestingly, however, Stathopoulou and colleagues (23) found that mood-induced opiate craving was not associated with self-reported opiate use to cope with negative affect. This finding is at odds with multiple studies that show that coping motives are associated with greater sensitivity to mood-induced drug-motivated behavior [Refs. (5, 7, 15, 16, 27, 37–43); but see Refs. (30, 40, 44)]. The fourth prediction of the current study, therefore, was that mood-induced heroin choice behavior would be greater in opiate users who reported using to cope with negative affect. Sensitivity to negative affect-triggered heroin seeking could be an important mechanism driving relapse (45, 46).

## Method

### Participants and Procedures

Participation was open to males and females aged 18–65 being treated for current heroin addiction by opioid medication at the Royal Prince Alfred (RPA) Hospital Drug Health Clinic in Sydney, Australia. Data were collected from 47 opiate-dependent outpatients (male = 32, female = 15) after they received opiate medication. In total, 2 participants (4.3%) were aged 19–24, 14 (29.8%) were 25–39, 16 (34.0%) were 40–49, and 15 participants (31.9%) were 50+ years of age. Thirty-five participants were receiving methadone (mean dose = 79 mg), 2 participants received buprenorphine (mean dose = 6 mg), and 10 participants were receiving suboxone (mean dose = 21 mg). The majority of these participants were currently unemployed, educated to high school level, and single. Eligibility criteria included: 1) current attendance in treatment for heroin addiction, 2) over 18 years of age, 3) English speaking, and 4) receiving opiate medication for the last 30 days. Healthy controls that did not have a history of opiate addiction were recruited *via* word of mouth from the community. Exclusion criteria included history of substance dependence or any other *DSM-IV* axis I disorders. Participants were matched for gender (opiate users = 33% female; controls = 48% female, Fishers exact *p* = .21), and age, *t*(34.26) = 1.66, *p* = .11. *A* chi-square comparing three categories of educational attainment (below high school, high school, greater than high school achievement) was non-significant, χ*^2^*(2, 71) = 4.48, *p* = .11, suggesting the two groups were matched for educational attainment. One opiate-using participant was excluded for showing an extremely outlying reduction in heroin choice from pre- to post-induction (>3 times the inter quartile range), leaving 46 opiate users and 25 control participants in the analyzed data set. The study was approved by the Western Sydney University Human Research Ethics Committee, and participants provided informed written consent.

### Questionnaires

Participants reported gender and age. Heroin users completed the Reasons for Drinking Questionnaire (RFDQ), adapted for heroin use (47). Instructions stated “The following 16 questions are a list of reasons why people take illicit opiates. Please rate each of these reasons on how important each is for you.” Within the questionnaire, the word “alcohol” from the original was replaced with the word “heroin.” Responses were scored on a 1–10 scale ranging from “not at all important” to “very important.” The RFDQ has three subscales reflecting heroin use to cope with negative affect, social pressure, and cued craving, obtained by averaging relevant items, giving a subscale score range of 1–10. We adapted the RFDQ because the drinking to cope subscale in the original version has been shown to be associated with greater sensitivity to negative mood-induced alcohol choice in two of our prior studies with student drinkers in a task similar to the present (27, 37).

### Mood-Induced Heroin Picture Choice Task

**Figure 1:**
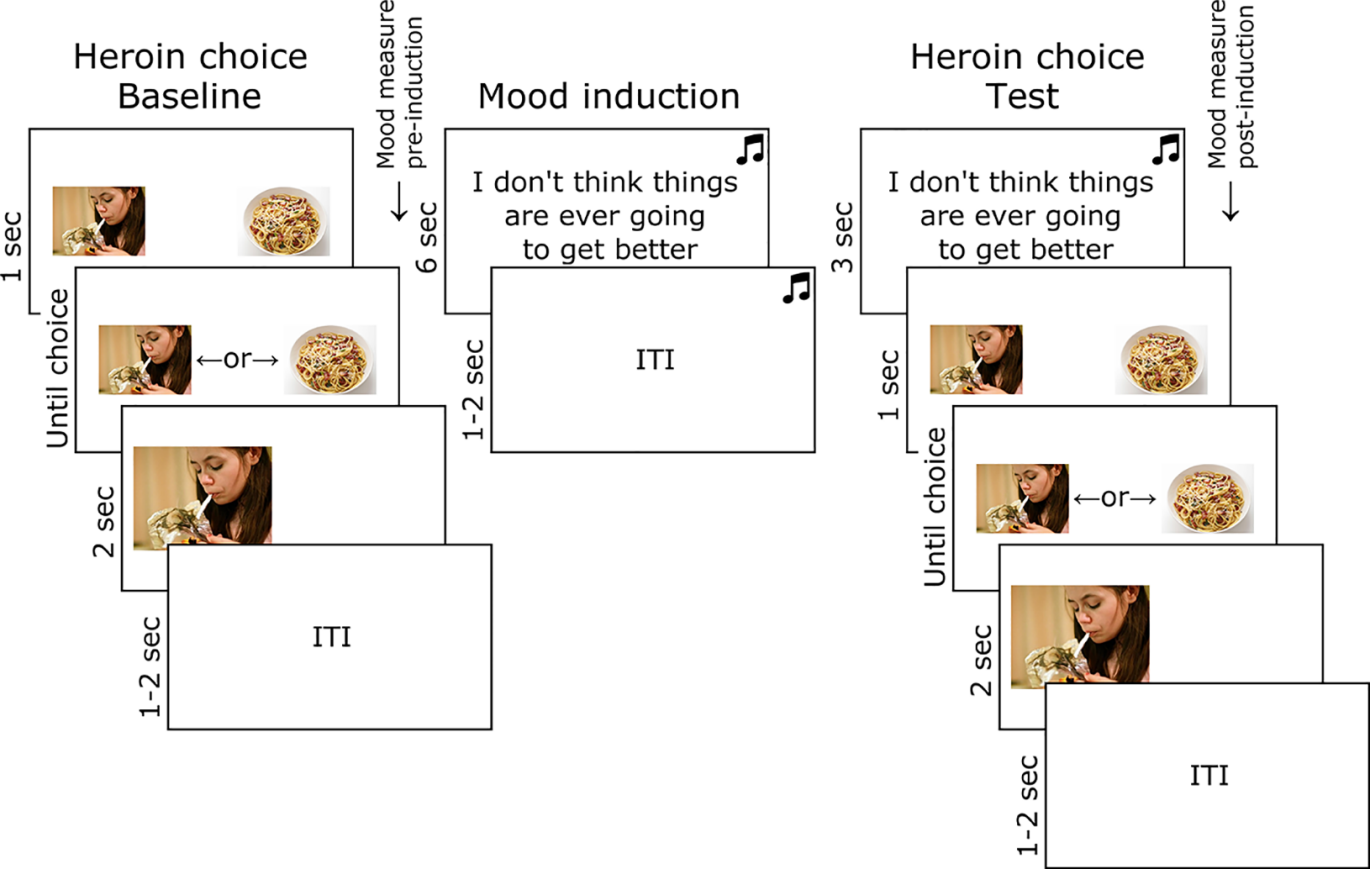
Task used to test the effect of mood induction on heroin choice in opiate-dependent and control participants. Images of heroin and food were obtained online and were not copyrighted.

The trial structure and timings of the heroin picture choice task are shown in **Figure 1**. At baseline, participants freely chose to enlarge a heroin or food thumbnail picture with a left or right key press, over 32 trials. In each trial, a heroin and food thumbnail was presented randomly in the left or right position, sampled from a set of 28 of each image type (obtained online from non-copyrighted images). Following baseline choice, pre-induction subjective mood was measured by participants reporting the extent to which they currently felt five negative (jittery, upset, distressed, sad, irritable) and five positive emotions (enthusiastic, happy, excited, inspired, alert), randomly ordered, on a five-point scale ranging from “not at all” to “extremely.” Sad music was then played through headphones (Barber’s Adagio for Strings), and participants were instructed to carefully consider 16 negative statements (e.g., “I don’t think things are ever going to get better”) randomly ordered [for full list, see Ref. (34)]. The heroin choice test comprised 32 trials identical to baseline, except that the sad music continued to play and a negative statement (randomly selected from the set of 16) was presented prior to each choice (the same picture set was used as at baseline). Post-induction subjective mood was then measured in the same way as before.

## Results

### Heroin Choice

**Figure 2:**
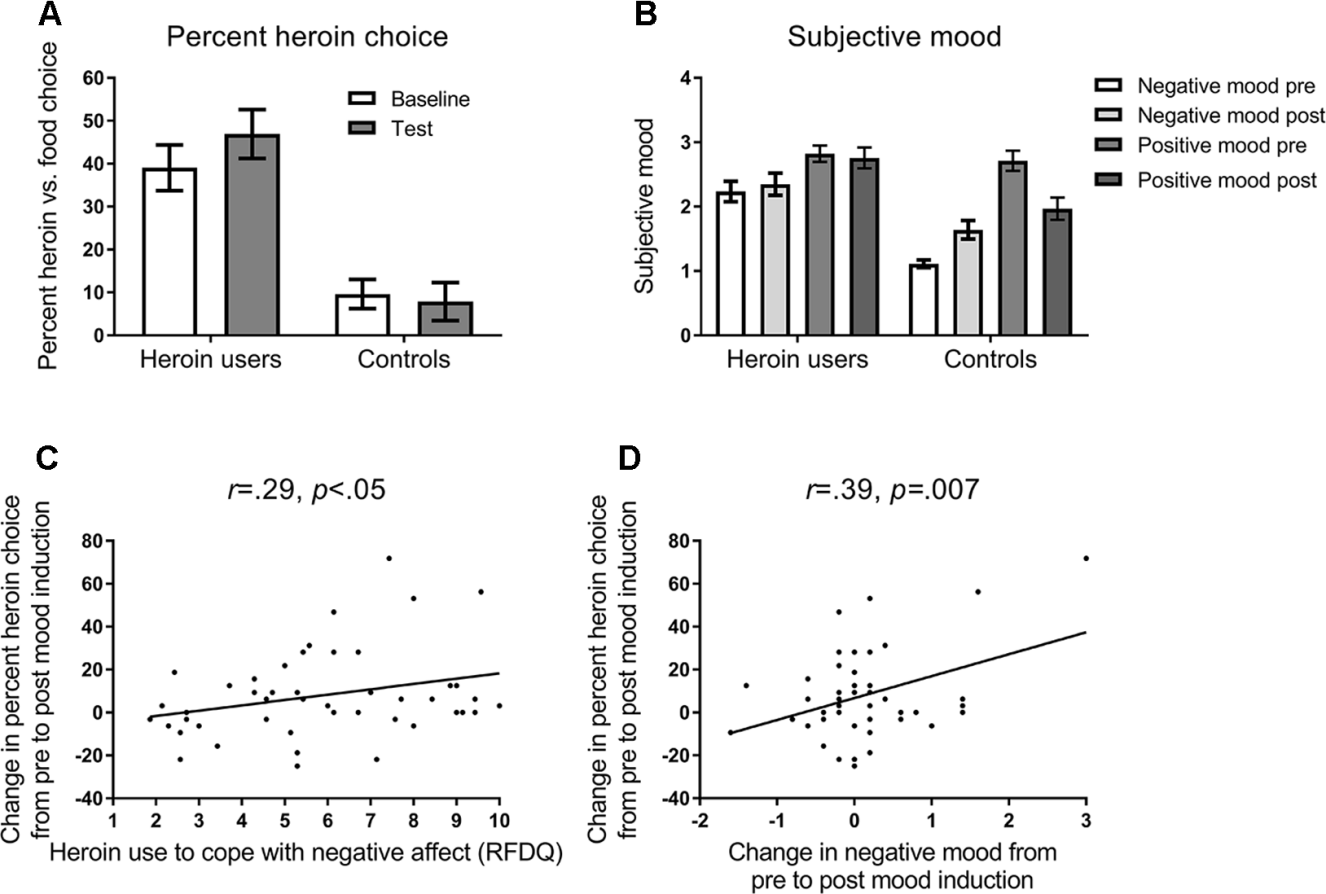
**(A)** Mean percent (and SEM) choice of heroin versus food pictures in the baseline and test blocks of the task (see **Figure 1**). Opiate-dependent participants showed a higher rate of heroin choice overall compared to control participants, and showed a mood induced increase in heroin choice at test, whereas controls did not. **(B)** Subjective negative and positive mood states reported at pre-induction and post-test timepoints (see **Figure 1**). Opiate-dependent participants showed no overall change in subjective mood states, whereas control participants showed an increase in negative mood and a decrease in positive mood following mood induction. **(C)** Scatterplot and regression slope relating the mood-induced change in percent heroin choice to self-reported opiate use to cope with negative affect in opiate-dependent participants. **(D)** Scatterplot and regression slope relating the mood-induced change in percent heroin choice to self-reported change in negative mood in opiate-dependent participants.

**Figure 2A** shows the percentage (and SEM) choice of heroin versus food images, in heroin users and controls. ANOVA on these data, with the variables group (heroin users, controls) and block (baseline, test), yielded a significant main effect of group, *F*(1,69) = 19.85, *p* = .001, η_p_^2^ = .223, and interaction between group and block, *F*(1,69) = 4.04, *p* = .048, η_p_^2^ = .055, and no significant main effect of block, *F*(1,69) = 1.64, *p* = .21, η_p_^2^ = .023. The main effect of block was significant in heroin users, *F*(1,45) = 6.96, *p* = .01, η_p_^2^ = .134, but not controls, *F*(1,24) = .26, *p* = .62, η_p_^2^ = .011. These results indicate that heroin users chose heroin images more frequently, and showed increased heroin choice following negative mood induction, compared to controls.

### Subjective Mood

**Figure 2B** shows the mean (and SEM) subjective negative and positive mood reported pre-induction and post-test. ANOVA on these data with the variables group (heroin users, controls), mood state (negative, positive), and timepoint (pre, post) yielded a significant interaction between group, mood state, and timepoint, *F*(1,69) = 12.40, *p* = .001, η_p_^2^ = .152. In heroin users, there was a significant main effect of mood state, *F*(1,45) = 6.06, *p* = .02, η_p_^2^ = .119, but no effect of timepoint, *F*(1,45) = .12, *p* = .73, η_p_^2^ = .003, or interaction between mood state and timepoint, *F*(1,45) = .91, *p* = .34, η_p_^2^ = .020. By contrast, in controls, there was a significant main effect of mood state, *F*(1,24) = 39.22, *p* < .001, η_p_^2^ = .620, and a significant interaction between mood state and timepoint, *F*(1,24) = 26.79, *p* < .001, η_p_^2^ = .528, with negative mood increasing significantly from pre to post, *F*(1,24) = 17.23, *p* < .001, η_p_^2^ = .419, and positive mood decreasing significantly from pre to post, *F*(1,24) = 16.06, *p* < .001, η_p_^2^ = .401. Thus, heroin users showed no change in subjective mood following mood induction, whereas controls showed the appropriate change in mood states.

### Correlations

As shown in **Figure 2C**, the change in heroin choice from the pre to post mood induction correlated positively with RFDQ heroin use to cope with negative affect, *r* = .29, *p* < .05, but not with RFDQ heroin use due to social pressure, *r* = −.13, *p* = .39, or cued craving, *r* = .10, *p* = .52. Furthermore, as shown in **Figure 2D**, heroin users’ change in heroin choice also correlated with the change in negative mood from pre- to post-induction, *r* = .39, *p* = .007, but not with the change in positive mood *r* = −.19, *p* = .20. RFDQ heroin use to cope with negative affect did not correlate significantly with the change in negative mood, *r* = .28, *p* < .06. Thus, heroin users’ change in heroin choice from baseline to test was amplified in those who reported heroin use to cope with negative affect, and those who reported the greatest increase in negative mood following induction.

## Discussion

The study found that opiate-dependent individuals chose heroin over food images more frequently than control participants. This accords with results from two studies with cocaine-dependent individuals, who chose cocaine over pleasant pictures more frequently than control participants (25, 26). Percent drug choice has also been shown to increase with dependence level within drug user groups (25–29). These findings suggest that the pictorial drug choice task is a valid assay of the relative value ascribed to the drug by drug users. The pictorial choice task may have the advantage over subjective craving as an outcome measure, in being more readily translatable to animal models that also use behavioral measures rather than subjective report (48, 49). This procedure also has an advantage over human concurrent drug self-administration procedures (50–52) in not requiring actual consumption, and so is technically simpler and ethically acceptable for clients who are currently abstinent. Finally, the pictorial drug choice task is superficially similar to attentional bias tasks, but appears more reliable in detecting group differences, and correlations with dependence severity (53).

The second finding was that negative mood induction increased heroin choice in opiate-dependent individuals but not control participants. This extends prior induction studies with opiate users (21–23), by including control participants to demonstrate the specificity of the mood induction effect. The finding also confirms that negative mood acts as a trigger for heroin-seeking behavior (and not just craving), as has been found with other drug classes including alcohol (4–7), tobacco (8–10), and cocaine (11–14).

Sensitivity to mood-induced heroin choice was also found to correlate with subjective changes in negative mood, consistent with two prior opiate studies (22, 23) and induction studies with other drug classes (27, 31–34). These findings accord with the prediction of affective negative reinforcement theory (54) in suggesting that the affective change produced by the induction procedure was responsible for the change in heroin-seeking behavior.

Finally, sensitivity to mood-induced heroin choice was found to correlate with self-reported opiate use to cope with negative affect, but not other opiate use motives (social pressure and cued craving). This finding contradicts the study by Stathopoulou and colleagues (23) which found no association between mood-induced opiate craving and opiate use to cope with negative affect, but corroborates multiple induction studies with other drug classes that have found this same association (5, 7, 15, 16, 27, 37–43). We may therefore accept our association as a true positive. It is possible that coping motives increase the risk of dependence by conferring sensitivity to negative affective triggers for drug-seeking behavior (45, 46).

We might further speculate that individual sensitivity to mood-induced heroin choice is a risk factor for relapse. The basis for this claim is that such sensitivity is associated with relapse risk in alcohol- (15–18) and cocaine-dependent individuals (19, 20). With respect to opiate users, poorer stress tolerance (55) and abnormal cortisol (56) predict poorer treatment engagement or earlier lapse, and preliminary evidence suggests that learning to cope with negative affect may promote abstinence (57). The implication is that sensitivity to negative mood-induced heroin-seeking is also a risk factor for relapse, and that treatments targeting this sensitivity may have efficacy for maintaining abstinence.

One limitation of the current study was that we did not observe an overall change in subjective mood following negative mood induction in the heroin user group, whereas controls did show changes to self-reported positive and negative mood. Despite this, the increase in heroin choice at test for the heroin user group, as well as the correlation between this effect and their change in negative mood, indicated that the mood induction procedure did impact the heroin user group. However, these effects were small and were perhaps reduced by the opiate replacement medications taken shortly before the experiment, similarly to acute alcohol, which has been shown to reduce mood induction effects (58). Future studies may employ a stronger mood induction procedure that produces a reliable change in subjective mood in heroin users, and a larger magnitude of effect on heroin choice behavior.

The second limitation was that we did not employ a control condition to determine whether the change in heroin picture choice was due to the mood induction or time. Previous studies have shown that drug choice remains stable over time then jumps following induction (30). Similarly, percent heroin choice in heroin users of the current study was stable across the two halves of the baseline phase (means = 39% and 39%, respectively), then jumped following induction and was stable across the two halves of the test phase (means = 48% and 46%, respectively). These data, plus the correlation between subjective mood and heroin choice, suggest that the increase in heroin choice was caused by negative mood induction and not by time.

The third limitation was that we could not obtain indices of psychiatric state in the two groups, because we had access to drug-using clients for an extremely short period during their hospital visit. As a consequence, we are unable to test whether the differential mood induction effect between the two groups was due to drug user status, or confounded psychiatric symptoms, such as anxiety or depression, which are known to be associated with greater sensitivity to mood induction effects on alcohol seeking (10, 27).

## Ethics Statement

This study was approved by the Western Sydney University Human Research Ethics Committee.

## Author Contributions

LHo designed the procedure and wrote the first draft of the manuscript. LHa and AB programmed the task and contributed to the analysis. JM and SC ran the participants and contributed to the analysis. GW and AM oversaw the running of the experiment. All authors contributed to the writing of the manuscript.

## Funding

LHo received an Alcohol Research UK grant (RS17/03).

## Conflict of Interest Statement

The authors declare that the research was conducted in the absence of any commercial or financial relationships that could be construed as a potential conflict of interest.

## References

[1] Hall FS Der-Avakian A Gould TJ Markou A Shoaib M Young JW Negative affective states and cognitive impairments in nicotine dependence. Neurosci Biobehav Rev (2015) 58:168–85. 10.1016/j.neubiorev.2015.06.004 PMC4670824 26054790

[2] Mathew AR Hogarth L Leventhal AM Cook JW Hitsman B Cigarette smoking and depression comorbidity: systematic review and proposed theoretical model. Addiction (2017) 112:401–12. 10.1111/add.13604 PMC5296249 27628300

[3] Crum RM Green KM Storr CL Chan YF Ialongo N Stuart EA Depressed mood in childhood and subsequent alcohol use through adolescence and young adulthood. Arch Gen Psychiatry (2008) 65:702–12. 10.1001/archpsyc.65.6.702 PMC4151267 18519828

[4] Amlung M MacKillop J Understanding the effects of stress and alcohol cues on motivation for alcohol *via* behavioral economics. Alcohol Clin Exp Res (2014) 38:1780–9. 10.1111/acer.12423 PMC4049358 24890323

[5] Rousseau GS Irons JG Correia CJ The reinforcing value of alcohol in a drinking to cope paradigm. Drug Alcohol Depend (2011) 118:1–4. 10.1016/j.drugalcdep.2011.02.010 21414732

[6] Zack M Poulos CX Fragopoulos F Woodford TM MacLeod CM Negative affect words prime beer consumption in young drinkers. Addict Behav (2006) 31:169–73. 10.1016/j.addbeh.2005.04.016 15922513

[7] Field M Quigley M Mild stress increases attentional bias in social drinkers who drink to cope: a replication and extension. Exp Clin Psychopharmacol (2009) 17:312–9. 10.1037/a0017090 19803630

[8] Heckman BW Carpenter MJ Correa JB Wray JM Saladin ME Froeliger B Effects of experimental negative affect manipulations on ad libitum smoking: a meta-analysis. Addiction (2015) 110:751–60. 10.1111/add.12866 PMC4398635 25641624

[9] Heckman BW Kovacs MA Marquinez NS Meltzer LR Tsambarlis ME Drobes DJ Influence of affective manipulations on cigarette craving: a meta-analysis. Addiction (2013) 108:2068–78. 10.1111/add.12284 PMC3830730 23795674

[10] Hogarth L Mathew AR Hitsman B Current major depression is associated with greater sensitivity to the motivational effect of both negative mood induction and abstinence on tobacco-seeking behavior. Drug Alcohol Depend (2017) 176:1–6. 10.1016/j.drugalcdep.2017.02.009 28460322 PMC5499379

[11] Sinha R Lacadie C Skudlarski P Fulbright RK Rounsaville BJ Kosten TR Neural activity associated with stress-induced cocaine craving: a functional magnetic resonance imaging study. Psychopharmacology (2005) 183:171–80. 10.1007/s00213-005-0147-8 16163517

[12] Sinha R Talih M Malison R Cooney N Anderson G Kreek M Hypothalamic–pituitary–adrenal axis and sympatho-adreno-medullary responses during stress-induced and drug cue-induced cocaine craving states. Psychopharmacology (2003) 170:62–72. 10.1007/s00213-003-1525-8 12845411

[13] Sinha R Fuse T Aubin LR O’Malley SS Psychological stress, drug-related cues and cocaine craving. Psychopharmacology (2000) 152:140–8. 10.1007/s002130000499 11057517

[14] Sinha R Catapano D O’Malley S Stress-induced craving and stress response in cocaine dependent individuals. Psychopharmacology (Berl) (1999) 142:343–51. 10.1007/s002130050898 10229058

[15] Brady KT Back SE Waldrop AE McRae AL Anton RF Upadhyaya HP Cold pressor task reactivity: predictors of alcohol use among alcohol-dependent individuals with and without comorbid posttraumatic stress disorder. Alcohol Clin Exp Res (2006) 30:938–46. 10.1111/j.1530-0277.2006.00097.x 16737451

[16] Cooney NL Litt MD Morse PA Bauer LO Gaupp L Alcohol cue reactivity, negative-mood reactivity, and relapse in treated alcoholic men. J Abnorm Psychol (1997) 106:243–50. 10.1037/0021-843X.106.2.243 9131844

[17] Higley A Crane N Spadoni A Quello S Goodell V Mason B Craving in response to stress induction in a human laboratory paradigm predicts treatment outcome in alcohol-dependent individuals. Psychopharmacology (2011) 218:121–9. 10.1007/s00213-011-2355-8 PMC3191263 21607563

[18] Sinha R Fox HC Hong K Hansen J Tuit K Kreek M Effects of adrenal sensitivity, stress- and cue-induced craving, and anxiety on subsequent alcohol relapse and treatment outcomes. Arch Gen Psychiatry (2011) 68:942–52. 10.1001/archgenpsychiatry.2011.49 PMC3668981 21536969

[19] Sinha R Garcia M Paliwal P Kreek M Rounsaville BJ Stress-induced cocaine craving and hypothalamic–pituitary–adrenal responses are predictive of cocaine relapse outcomes. Arch Gen Psychiatry (2006) 63:324–31. 10.1001/archpsyc.63.3.324 16520439

[20] Back SE Hartwell K DeSantis SM Saladin M Rae-Clark AL Price KL Reactivity to laboratory stress provocation predicts relapse to cocaine. Drug Alcohol Depend (2010) 106:21–7. 10.1016/j.drugalcdep.2009.07.016 PMC2815094 19726138

[21] Childress AR Ehrman R McLellan AT MacRae J Natale M O’Brien CP Can induced moods trigger drug-related responses in opiate abuse patients? J Subst Abuse Treat (1994) 11:17–23. 10.1016/0740-5472(94)90060-4 8201629

[22] Hyman SM Fox H Hong KA Doebrick C Sinha R Stress and drug-cue-induced craving in opioid-dependent individuals in naltrexone treatment. Exp Clin Psychopharmacol (2007) 15:134–43. 10.1037/1064-1297.15.2.134 PMC2392893 17469937

[23] Stathopoulou G Pollack MH Otto MW Anxiety sensitivity moderates drug cravings in response to induced negative affect in opioid dependent outpatients. Addict Behav (2018) 84:75–8. 10.1016/j.addbeh.2018.03.020 29631093

[24] Tiffany ST A cognitive model of drug urges and drug-use behaviour: role of automatic and nonautomatic processes. Psychol Rev (1990) 97:147–68. 10.1037/0033-295X.97.2.147 2186423

[25] Moeller SJ Beebe-Wang N Woicik PA Konova AB Maloney T Goldstein RZ Choice to view cocaine images predicts concurrent and prospective drug use in cocaine addiction. Drug Alcohol Depend (2013) 130:178–85. 10.1016/j.drugalcdep.2012.11.001 PMC3609942 23218913

[26] Moeller SJ Maloney T Parvaz MA Dunning JP Alia-Klein N Woicik PA Enhanced choice for viewing cocaine pictures in cocaine addiction. Biol Psychiatry (2009) 66:169–76. 10.1016/j.biopsych.2009.02.015 PMC2742172 19358975

[27] Hogarth L Hardy L Mathew AR Hitsman B Negative mood-induced alcohol-seeking is greater in young adults who report depression symptoms, drinking to cope, and subjective reactivity. Exp Clin Psychopharmacol (2018) 26:138–46. 10.1037/pha0000177 PMC5896502 29389212

[28] Hardy L Parker S Hartley L Hogarth L A concurrent pictorial drug choice task marks multiple risk factors in treatment-engaged smokers and drinkers. Behav Pharmacol (2018) 29:716–25. 10.1097/fbp.0000000000000421 30169375

[29] Miele A Thompson M Jao NC Kalhan R Leone F Hogarth L Cancer patients enrolled in a smoking cessation clinical trial: characteristics and correlates of smoking rate and nicotine dependence. J Addict (2018) 2018:7. 10.1155/2018/2438161 PMC5846375 29682394

[30] Hardy L Hogarth L A novel concurrent pictorial choice model of mood-induced relapse in hazardous drinkers. Exp Clin Psychopharmacol (2017) 25:448–55. 10.1037/pha0000155 29251973

[31] Owens MM Ray LA MacKillop J Behavioral economic analysis of stress effects on acute motivation for alcohol. J Exp Anal Behav (2015) 103:77–86. 10.1002/jeab.114 25413719

[32] Sinha R Fox HC Hong KA Bergquist K Bhagwagar Z Siedlarz KM Enhanced negative emotion and alcohol craving, and altered physiological responses following stress and cue exposure in alcohol dependent individuals. Neuropsychopharmacology (2009) 34:1198–208. 10.1038/npp.2008.78 PMC2734452 18563062

[33] Kelly AB Masterman PW Young RM Negative mood, implicit alcohol-related memory, and alcohol use in young adults: the moderating effect of alcohol expectancy. Addict Behav (2011) 36:148–51. 10.1016/j.addbeh.2010.08.025 20869814

[34] Hogarth L He Z Chase HW Wills AJ Troisi J II Leventhal AM Negative mood reverses devaluation of goal-directed drug-seeking favouring an incentive learning account of drug dependence. Psychopharmacology (2015) 232:3235–47. 10.1007/s00213-015-3977-z PMC4534490 26041336

[35] Magrys SA Olmstead MC Acute stress increases voluntary consumption of alcohol in undergraduates. Alcohol Alcohol (2015) 50:213–8. 10.1093/alcalc/agu101 25557606

[36] McGrath E Jones A Field M Acute stress increases ad-libitum alcohol consumption in heavy drinkers, but not through impaired inhibitory control. Psychopharmacology (2016) 233:1227–34. 10.1007/s00213-016-4205-1 PMC4801987 26815361

[37] Hogarth L Hardy L Depressive statements prime goal-directed alcohol-seeking in individuals who report drinking to cope with negative affect. Psychopharmacology (2018) 235:269–79. 10.1007/s00213-017-4765-8 PMC5748391 29082424

[38] Birch CD Stewart SH Wall A McKee SA Eisnor SJ Theakston JA Mood-induced increases in alcohol expectancy strength in internally motivated drinkers. Psychol Addict Behav (2004) 18:231–8. 10.1037/0893-164X.18.3.231 15482078

[39] Grant VV Stewart SH Birch CD Impact of positive and anxious mood on implicit alcohol-related cognitions in internally motivated undergraduate drinkers. Addict Behav (2007) 32:2226–37. 10.1016/j.addbeh.2007.02.012 17408867

[40] Field M Powell H Stress increases attentional bias for alcohol cues in social drinkers who drink to cope. Alcohol Alcohol (2007) 42:560–6. 10.1093/alcalc/agm064 17766316

[41] Zack M Poulos CX Fragopoulos F MacLeod CM Effects of negative and positive mood phrases on priming of alcohol words in young drinkers with high and low anxiety sensitivity. Exp Clin Psychopharmacol (2003) 11:176–85. 10.1037/1064-1297.11.2.176 12755462

[42] Austin JL Smith JE Drinking for negative reinforcement: the semantic priming of alcohol concepts. Addict Behav (2008) 33:1572–80. 10.1016/j.addbeh.2008.07.016 18778899

[43] Woud ML Becker ES Rinck M Salemink E The relationship between drinking motives and alcohol-related interpretation biases. J Behav Ther Experimental Psychiatry (2015) 47:102–10. 10.1016/j.jbtep.2014.11.012 25525773

[44] Thomas SE Merrill JE von Hofe J Magid V Coping motives for drinking affect stress reactivity but not alcohol consumption in a clinical laboratory setting. J Studies Alcohol Drugs (2014) 75:115–23. 10.15288/jsad.2014.75.115 PMC3893625 24411803

[45] Crum RM Mojtabai R Lazareck S Bolton JM Robinson J Sareen J A prospective assessment of reports of drinking to self-medicate mood symptoms with the incidence and persistence of alcohol dependence. JAMA Psychiatry (2013) 70:718–26. 10.1001/jamapsychiatry.2013.1098 PMC4151472 23636710

[46] Crum RM La Flair L Storr CL Green KM Stuart EA Alvanzo AAH Reports of drinking to self-medicate anxiety symptoms: longitudinal assessment for subgroups of individuals with alcohol dependence. Depress Anxiety (2013) 30:174–83. 10.1002/da.22024 PMC4154590 23280888

[47] Zywiak WH Connors GJ Maisto SA Westerberg VS Relapse research and the Reasons for Drinking Questionnaire: a factor analysis of Marlatt's relapse taxonomy. Addiction (1996) 91:121–30. 10.1046/j.1360-0443.91.12s1.2.x 8997786

[48] Russo M Funk D Loughlin A Coen K Lê AD Effects of alcohol dependence on discrete choice between alcohol and saccharin. Neuropsychopharmacology (2018) 43:1859–66. 10.1038/s41386-018-0101-1 PMC6046058 29875449

[49] Guillem K Brenot V Durand A Ahmed SH Neuronal representation of individual heroin choices in the orbitofrontal cortex. Addict Biol (2018) 23:880–8. 10.1111/adb.12536 28703355

[50] Bickel WK DeGrandpre RJ Higgins ST Hughes JR Badger GJ Effects of simulated employment and recreation on drug taking: a behavioral economic analysis. Exp Clin Psychopharmacol (1995) 3:467–76. 10.1037/1064-1297.3.4.467

[51] Hart CL Haney M Foltin RW Fischman MW Alternative reinforcers differentially modify cocaine self-administration by humans. Behav Pharmacol (2000) 11:87–91. 10.1097/00008877-200002000-00010 10821213

[52] Stoops WW Lile JA Glaser PEA Hays LR Rush CR Alternative reinforcer response cost impacts cocaine choice in humans. Prog Neuropsychopharmacol Biol Psychiatry (2012) 36:189–93. 10.1016/j.pnpbp.2011.10.003 PMC3229673 22015480

[53] Field M Werthmann J Franken I Hofmann W Hogarth L Roefs A The role of attentional bias in obesity and addiction. Health Psychol (2016) 35:767–80. 10.1037/hea0000405 27505196

[54] Baker TB Piper ME McCarthy DE Majeskie MR Fiore MC Addiction motivation reformulated: an affective processing model of negative reinforcement. Psychol Rev (2004) 111:33–51. 10.1037/0033-295X.111.1.33 14756584

[55] Strong DR Brown RA Sims M Herman DS Anderson BJ Stein MD Persistence on a stress-challenge task before initiating buprenorphine treatment was associated with successful transition from opioid use to early abstinence. J Addict Med (2012) 6:219–25. 10.1097/ADM.0b013e31825d927f PMC3980199 22864399

[56] Jaremko KM Sterling RC Van Bockstaele EJ Psychological and physiological stress negatively impacts early engagement and retention of opioid-dependent individuals on methadone maintenance. J Subst Abuse Treat (2015) 48:117–27. 10.1016/j.jsat.2014.08.006 PMC4250337 25239858

[57] Stein MD Herman DS Moitra E Hecht J Lopez R Anderson BJ A preliminary randomized controlled trial of a distress tolerance treatment for opioid dependent persons initiating buprenorphine. Drug Alcohol Depend (2015) 147:243–50. 10.1016/j.drugalcdep.2014.11.007 PMC4297723 25510307

[58] Kushner MG Mackenzie TB Fiszdon J Valentiner DP Foa E Anderson N The effects of alcohol consumption on laboratory-induced panic and state anxiety. Arch Gen Psychiatry (1996) 53:264–70. 10.1001/archpsyc.1996.01830030086013 8611064

